# Enhanced recovery after surgery protocol and postoperative opioid prescribing for cesarean delivery: an interrupted time series analysis

**DOI:** 10.1186/s13741-021-00209-0

**Published:** 2021-11-15

**Authors:** E. M. Langnas, Z. A. Matthay, A. Lin, M. W. Harbell, R. Croci, R. Rodriguez-Monguio, C. L. Chen

**Affiliations:** 1 Department of Anesthesia and Perioperative Care, University of California, San Francisco, 513 Parnassus Ave, S455, San Francisco, CA 94143 USA; 2 Department of Surgery, University of California, San Francisco, San Francisco, USA; 3 UCSF School of Medicine, University of California, San Francisco, San Francisco, USA; 4 Department of Clinical Pharmacy, University of California, San Francisco, San Francisco, USA; 5 Medication Outcomes Center, University of California, San Francisco, San Francisco, USA; 6 Philip R. Lee Institute for Health Policy Studies at the University of California, San Francisco, San Francisco, USA; 7 Department of Anesthesiology and Perioperative Medicine, Mayo Clinic, 5777 E Mayo Blvd, Phoenix, AZ 85054 USA; 8 UCSF Health Informatics, University of California, San Francisco, San Francisco, USA

**Keywords:** Opioids, Cesarean delivery, Multimodal analgesia, Oral morphine equivalents, Postoperative pain

## Abstract

**Introduction:**

Enhanced recovery after surgery (ERAS) pathways have emerged as a promising strategy to reduce postoperative opioid use and decrease the risk of developing new persistent opioid use in surgical patients. However, the association between ERAS implementation and discharge opioid prescribing practices is unclear.

**Study design:**

We conducted a retrospective observational quasi-experimental study of opioid-naïve patients aged 18+ undergoing cesarean delivery between February 2015 and December 2019 at a large academic center. An interrupted time series analysis (ITSA) was used to model the changes in pain medication prescribing associated with the implementation of ERAS to account for pre-existing temporal trends.

**Results:**

Among the 1473 patients (out of 2249 total) who underwent cesarean delivery after ERAS implementation, 80.72% received a discharge opioid prescription vs. 95.36% at baseline. Pre-ERAS daily oral morphine equivalents (OME) on the discharge prescription decreased by 0.48 OME each month (*p*<0.01). There was a level shift of 35 more OME prescribed (*p*<0.01), followed by a monthly decrease of 1.4 OMEs per month after ERAS implementation (*p*<0.01). Among those who received a prescription, 61.35% received a total daily dose greater than 90 OME compared to 11.35% pre-implementation (*p*<0.01), while prescriptions with a total daily dose less than 50 OME decreased from 79.86 to 25.85% after ERAS implementation(*p*<0.01).

**Conclusion:**

Although ERAS implementation reduced the overall proportion of patients receiving a discharge opioid prescription after cesarean delivery, for the subset of patients receiving an opioid prescription, ERAS implementation may have inadvertently increased the prescribing of daily doses greater than 90 OME. This finding highlights the importance of early and continued evaluation after new policies are implemented.

## Introduction

In the USA, opioids are often over-prescribed to patients after surgical procedures, which results in excess and unused opioids (Berterame et al. 2016; Baker et al. 2016; Wunsch et al. 2016; Bicket et al. 2017; Brummett et al. 2019; Howard et al. 2019). Excess opioids prescribed at discharge are a potential source for overdose, misuse, diversion, and new persistent opioid use among surgical patients (Berterame et al. 2016; Dowell et al. 2013; Brummett et al. 2017). Specialty guidelines and consensus statements have emerged to address the need for providing adequate pain control while mitigating the risk of opioid-related adverse effects (American Society of Anesthesiologists Task Force on Acute Pain Management 2012; Levy et al. 2021).

Cesarean delivery is the most common inpatient surgical procedure performed in the USA with approximately 1.3 million cesarean deliveries performed annually (Martin et al. 2018). Almost all women undergoing cesarean delivery are exposed to opioids during their hospitalization, and 85% of women fill their first opioid prescription upon hospital discharge (Bateman et al. 2017). Between 0.4 and 2.2% of women who received an opioid prescription after cesarean delivery become persistent opioid users (Bateman et al. 2017; Peahl et al. 2019). Furthermore, 83% of women undergoing cesarean delivery reported having leftover prescription opioids, which may increase the risk of misuse or diversion (Bartels et al. 2016).

Enhanced Recovery After Surgery (ERAS) pathways have emerged as a promising strategy to reduce opioid consumption after surgery (Wick et al. 2017). ERAS pathways utilize a standardized multimodal analgesic regimen that includes non-opioid analgesics to minimize perioperative opioid use. ERAS has been effective in reducing inpatient opioid consumption and discharge opioid prescriptions in a variety of surgical procedures (Page et al. 2016; Talboys et al. 2016; Sarin et al. 2016; Chapman et al. 2016; Liu et al. 2020; Hedderson et al. 2019). However, it is unclear how implementation of standardized protocols impacts provider prescribing habits. Prior studies on ERAS implementation that utilize pre- and post-cohort analyses do not necessarily account for longitudinal temporal trends in the data, since all of the data is essentially clustered and analyzed within the two groups.

To examine the association between the ERAS implementation for cesarean delivery and opioid prescribing patterns over time, we conducted an interrupted time series analysis at a large academic medical center using electronic medical record (EMR) data. Our secondary aim was to evaluate trends in pain medication administration 24 h prior to discharge, as well as the trends in opioid refills within 90 days of discharge.

## Methods

### Study design and data source

We conducted a single-center, retrospective, observational quasi-experimental study of opioid-naïve patients aged 18+ undergoing cesarean delivery from February 2015 through December 2019 at the University of California San Francisco Medical Center (UCSF). This study was approved by the UCSF IRB, which waived patient consent for acquisition of data (IRB# 18-26728). Data was obtained by retrospective database queries of the UCSF electronic medical record (Epic Systems, Verona, WI).

After extraction from an electronic data warehouse, the data were validated for accuracy with iterative chart auditing. To ensure accurate and complete data extraction, data reports were evaluated to identify inconsistencies, missingness, extreme values, and invalid codes. Discrepancy management included reviewing discrepancies, investigating the reason, and resolving them. The data extracted had no missingness. After a proper quality check and assurance, the final dataset was locked so that the dataset could not be modified and only the final clean dataset was used for analysis.

### Study cohort

Our study included opioid-naïve patients aged 18 years and older who underwent elective or non-elective cesarean delivery and who were discharged to either home, a skilled nursing facility, or a rehabilitation facility. We defined opioid-naïve as any patient without an active opioid prescription documented in their electronic medical record (EMR) starting 6 months prior to hospital admission.

### Enhanced recovery after surgery

After multidisciplinary involvement from obstetrics, anesthesiology, pediatrics and lactation, an ERAS protocol for cesarean delivery was implemented for elective cesarean delivery starting on September 1, 2016, and was expanded to all cesarean deliveries starting on February 1, 2017. Prior to implementation, anesthesiology and obstetric attending physicians, residents, nurses, and mid-wives underwent training and education of the ERAS pathway, and order sets were created in the EMR to facilitate compliance with the ERAS pathway. The institutional ERAS protocol for inpatient pain management for cesarean delivery recommended oral acetaminophen 1000 mg every 8 h, intravenous ketorolac 30 mg every 8 h for 3 doses followed by oral ibuprofen 600 mg every 6 h, and oral oxycodone 5–10 mg as needed for moderate pain. Patients could receive hydromorphone 0.2–0.6 mg intravenously (IV) as needed for severe pain, but only after evaluation by an anesthesia provider. The default order set for discharge medications included acetaminophen 1000 mg every 8 h, ibuprofen 600 mg every 6 h, and oxycodone 5 to 10mg every 4 h as needed for a maximum of twenty pills without any refills.

### Opioid dose calculation

To compare pre- and post-discharge opioid administration, we converted all IV, oral, regional, and neuraxial opioid consumed in the 24 h leading up to hospital discharge into oral morphine equivalents (OME) using the 2018 UCSF Pain Management Committee’s opioid equivalence equation (University of California San Francisco, Pain Management Committee’s 2018). The opioid dosage on the discharge opioid prescription was also converted into OMEs using the same opioid conversion equation. The daily dose on the discharge opioid prescription was defined as the maximum allowable dose in a 24 h period according to the written prescription.

### Definition of high-risk prescription

The risk of opioid-related adverse events is associated with the maximum daily OME prescribed (Bohnert et al. 2011; Brat Gabriel et al. 2018). Consistent with CDC recommendations, we defined a high-risk prescription as a discharge opioid prescription exceeding 90 OME per day, which has been associated with an increased risk of opioid-related adverse effects, including overdose death (Dowell et al. 2016).

### Covariates

We assessed other variables that may be associated with the opioid discharge prescription, including patient demographic characteristics, history of substance use disorder diagnosis, depression or anxiety diagnosis, discharge service, and hospital length of stay (LOS).

### Statistical analysis

Data were summarized using mean and standard deviations for continuous and normally distributed variables, median and interquartile ranges for non-normally distributed variables, and as percentages for binary variables. Chi-squared tests were used to compare patient characteristics pre- and post-ERAS implementation.

An interrupted time series with segmented regression analysis (ITSA) was used to model the changes in pain medication prescribing practices associated with the implementation of ERAS. All ITSAs were performed using the ordinary least squares method with Newey-West standard errors (Linden 2015). A Cumby-Huizinga test was used to assess temporal autocorrelation, and standard error adjustments were incorporated for up to a 12-month lag in our models. As a sensitivity analysis, we examined whether patient characteristics (age and length of stay) or prescribing provider type (trainee or clinical nurse midwife) affected the models when included as covariates (Table 3 in Appendix). None of these significantly altered the association of ERAS with the pain medication outcome variables and therefore were not included in the final models. All data analyses were performed in Stata (Version 15, Stata Corps).

## Results

A total of 2249 opioid-naïve patients underwent cesarean delivery during the study period. We found significant differences in opioid use and prescribing practices pre- and post-ERAS implementation (Table 1). Post-ERAS, 49.76% of patients did not require opioids in the 24 h prior to discharge, compared to 21.01% pre-ERAS (*p*<0.01). In addition, 80.72% of patients were discharged with an opioid prescription post-ERAS implementation compared to 95.35% of patients pre-ERAS implementation (*p*<0.01). Among those who received a prescription, 96.55% of post-ERAS opioid type was oxycodone, compared to 17.03% pre-ERAS (*p*<0.01). Post-ERAS 61.35% received a total daily dose greater than 90 OME compared to 11.35% pre-ERAS (*p*<0.01), while prescriptions with a total daily dose less than 50 OME decreased from 79.86 to 25.85% after ERAS implementation(*p*<0.01). In addition, post-ERAS, we found a reduction in the mean days’ supply on the discharge prescription (5.48 days vs 2.23 days, *p*<0.01). There was no significant change in postoperative length of stay post-ERAS (Figure 4 in Appendix).

**Table 1:** Demographic and clinical characteristics of patients pre- and post-ERAS implementation

	Pre-ERAS (*n* = 776)		Post-ERAS (*n* = 1473)		*P* value
	*n*	%	*n*	%	
Age					
18–24	37	4.77%	75	5.09%	0.04
25–34	359	46.26%	603	40.94%	
35–44	366	47.16%	749	50.85%	
>44	14	1.80%	46	3.12%	
Race					
White	347	44.72%	677	45.96%	0.38
Asian	180	23.20%	334	22.74%	
Black or African American	49	6.31%	87	5.91%	
Native Hawaiian or Other Pacific Islander	16	2.06%	19	1.29%	
American Indian or Alaska Native	4	0.52%	8	0.54%	
Unknown	180	23.20%	348	23.56%	
Comorbidities					
Anxiety	3	0.39%	4	0.27%	0.64
Depression	50	6.44%	111	7.54%	0.33
Substance use disorder	5	0.64%	13	0.88%	0.54
Opioid-free 24 h pre-discharge	163	21.01%	733	49.76%	<0.01
Discharged with opioid prescription	740	95.36%	1189	80.72%	<0.01
Daily oral morphine equivalents on discharge prescription					
>0–49	591	79.86%	338	28.45%	<0.01
50–89	65	8.78%	119	10.02%	
90 or more	84	11.35%	731	61.53%	
Proportion of discharge prescriptions that are oxycodone	126	17.03%	1148	96.55%	<0.01
Days of opioid prescription (mean, standard deviation)	5.48, 2.05		2.23, 1.95		<0.01

**Fig. 1:**
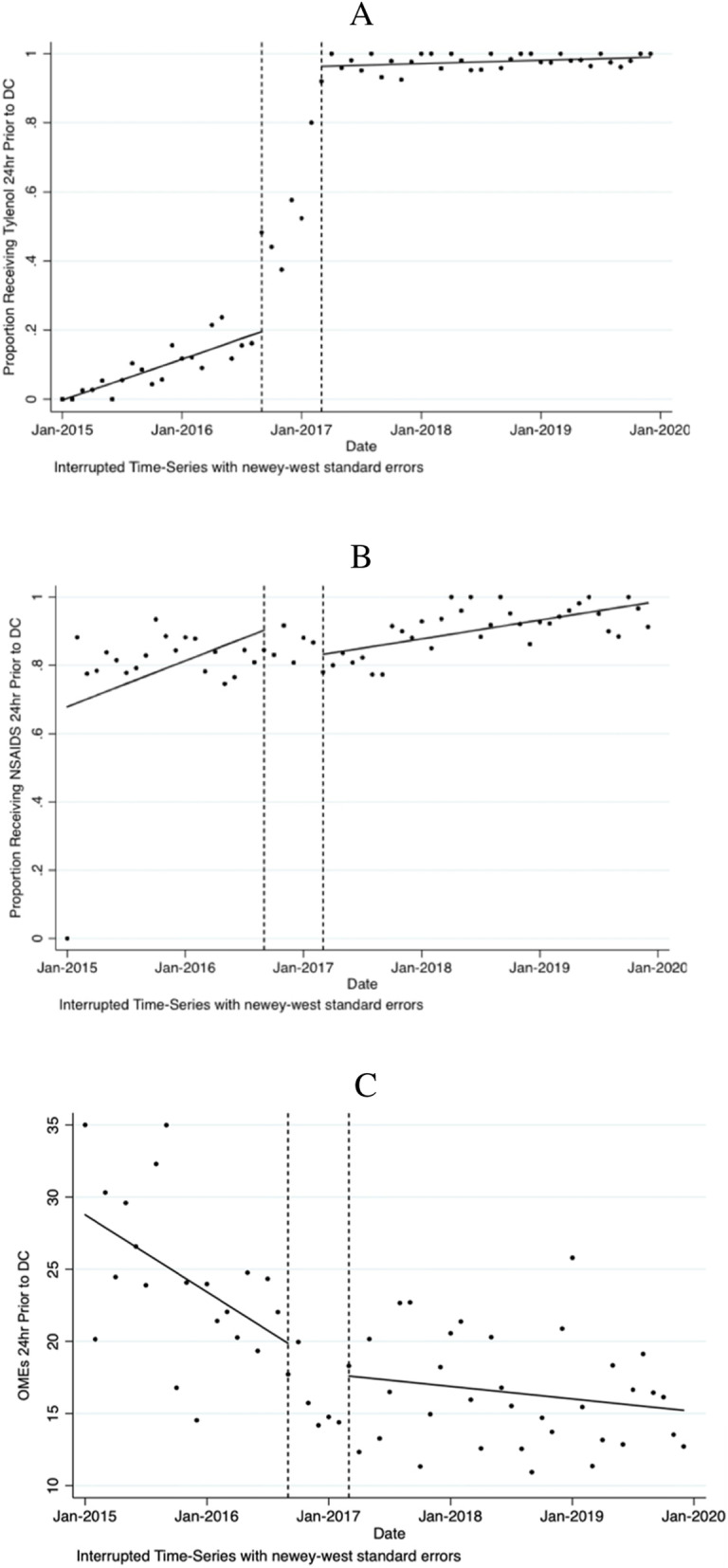
Changes in medications consumed 24 h prior to discharge, 2015–2019. Data are presented in monthly intervals on the *x*-axis. Vertical dashed lines represent the implementation phase. DC = discharge; OME = oral morphine equivalent; ERAS = Enhanced recovery after surgery; NSAID = non-steroidal anti-inflammatory. **a** Proportion of patients receiving acetaminophen in the 24 h prior to discharge. Post-ERAS associated with a level increase of 77% (*p*<0.01). **b** Proportion of patients receiving NSAID in the 24 h prior to discharge. No significant change was noted post-ERAS. **c** Mean oral morphine equivalents administered 24 h prior to discharge. Post-ERAS level change of 2.7 fewer OME (*p*=0.06).

An interrupted time series analysis was used to evaluate the association of ERAS on pain medication consumption in the 24 h prior to discharge (Fig. 1, Table 2). There was a statistically significant increase (77%) in acetaminophen consumption 24 h prior to discharge immediately following ERAS implementation (*p*<0.01) (Fig. 1a). In contrast, there was no significant change in the consumption of NSAIDs prior to discharge (Fig. 1b).

**Table 2:** Interrupted time series analysis examining pain medication utilization pre- and post-intervention

	Coefficient	95% CI low	95% CI high	***P*** value
**Proportion prescribed tylenol 24 h prior to discharge**				
Pre-ERAS trend (Jan 2015–Sep 2016)	0.01	0.01	0.01	<0.01
Level shift after ERAS implementation	0.77	0.75	0.78	<0.01
Trend shift after ERAS implementation	− 0.01	− 0.01	− 0.01	<0.01
Post-ERAS trend (Feb 2017–Dec 2019)	0.00	0.00	0.00	<0.01
**Proportion prescribed NSAIDs 24 h prior to discharge**				
Pre-ERAS trend (Jan 2015–Sep 2016)	0.01	0.00	0.03	0.12
Level shift after ERAS implementation	− 0.07	− 0.20	0.05	0.25
Trend shift after ERAS implementation	− 0.01	− 0.02	0.01	0.38
Post-ERAS trend (Feb 2017–Dec 2019)	0.00	0.00	0.01	<0.01
**Mean OME administered in 24 h prior to discharge**				
Pre-ERAS trend (Jan 2015–Sep 2016)	− 0.45	− 0.60	− 0.29	<0.01
Level shift after ERAS implementation	− 2.27	− 4.64	0.10	0.06
Trend shift after ERAS implementation	0.37	0.22	0.53	<0.01
Post-ERAS trend (Feb 2017–Dec 2019)	− 0.07	− 0.13	− 0.02	0.01
**Total OME prescribed at discharge**				
Pre-ERAS trend (Jan 2015–Sep 2016)	− 2.26	− 3.54	− 0.98	<0.01
Level shift after ERAS implementation	− 51.50	− 95.97	− 7.04	0.02
Trend shift after ERAS implementation	− 1.52	− 3.34	0.29	0.10
Post-ERAS trend (Feb 2017–Dec 2019)	− 3.78	− 5.59	− 1.97	<0.01
**Daily OME prescribed at discharge**				
Pre-ERAS trend (Jan 2015–Sep 2016)	− 0.48	− 0.75	− 0.22	<0.01
Level shift after ERAS implementation	34.83	29.13	40.53	<0.01
Trend shift after ERAS implementation	− 0.89	− 1.22	− 0.57	<0.01
Post-ERAS trend (Feb 2017–Dec 2019)	− 1.38	− 1.62	− 1.13	<0.01
**Proportion prescribed opioid refill within 90 days after discharge**				
Pre-ERAS trend (Jan 2015–Sep 2016)	− 0.03%	− 0.25%	0.18%	0.77
Level shift after ERAS implementation	− 4.96%	− 9.68%	− 0.24%	0.04
Trend shift after ERAS implementation	0.01%	− 0.23%	0.26%	0.91
Post-ERAS trend (Feb 2017–Dec 2019)	− 0.02%	− 0.19%	0.15%	0.85

*OME* oral morphine equivalents, *ERAS* enhanced recovery after surgery, *NSAIDS* non-steroidal anti-inflammatory drugs, *CI* confidence interval

Pre-ERAS opioid consumption in the 24 h prior to discharge showed a month-to-month reduction of 0.48 OME (*p*<0.01) (Fig. 1c). There was a post-intervention level change of 2.7 fewer OMEs prescribed which was approaching significance (*p*=0.06), followed by a post-intervention monthly reduction of 0.07 OMEs (*p*=0.01) (Fig. 1c).

**Fig. 2:**
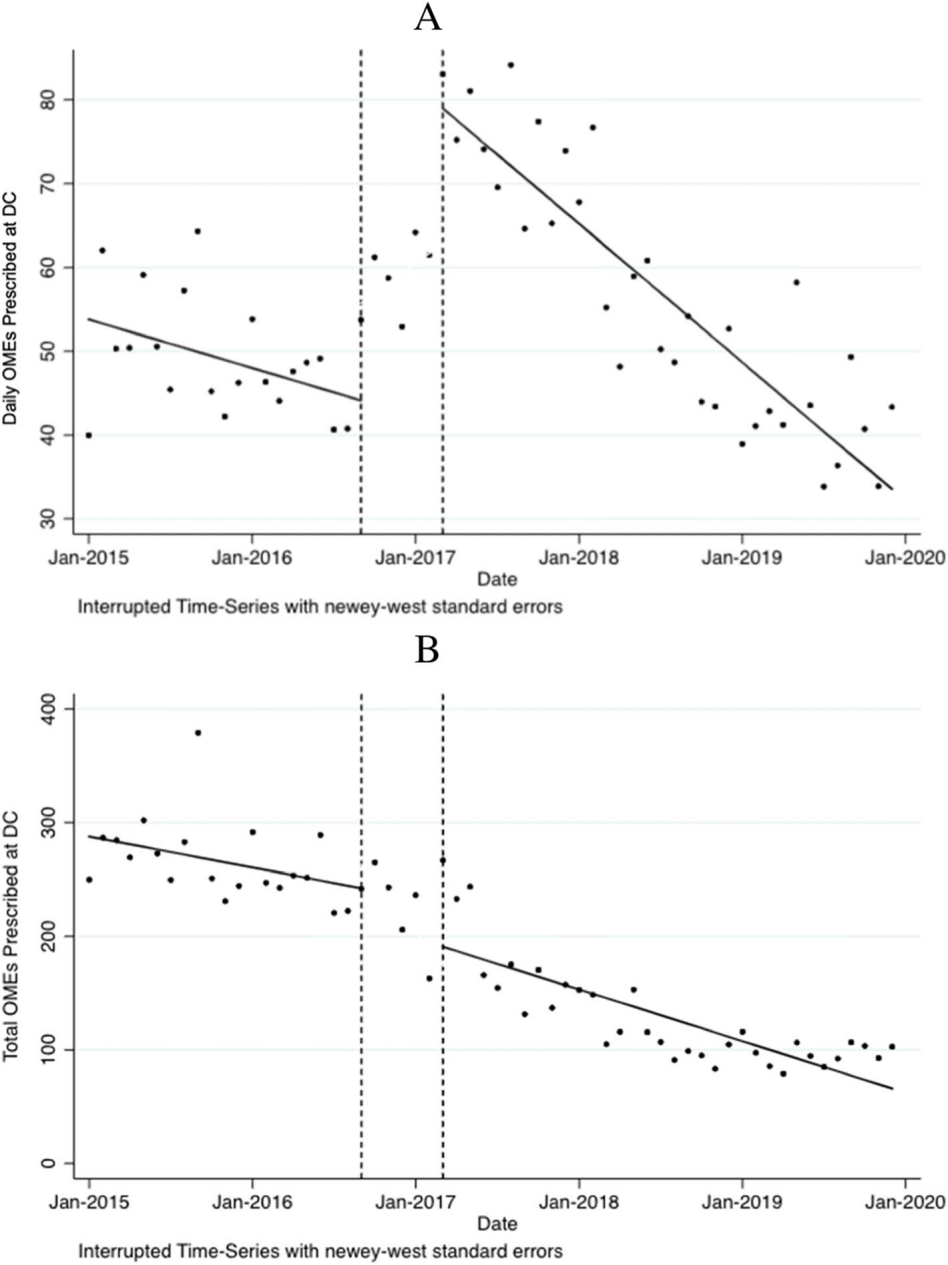
Change in total and daily oral morphine equivalents on the discharge opioid prescription, 2015–2019. Data are presented in monthly intervals on the *x*-axis and oral morphine equivalents on the *y*-axis. Vertical dashed lines represent the implementation phase. DC = discharge; OME = oral morphine equivalent; ERAS = Enhanced recovery after surgery. **a** Changes in mean daily OME on the discharge prescription. Post-ERAS implementation associated with a level change of 35 more daily OME prescribed (*p*<0.01). **b** Changes in total OME on discharge prescription. Post-ERAS implementation associated with a level change of 51.5 fewer OME prescribed (*p*<0.05)

Pre-ERAS daily OMEs on the opioid discharge prescription showed a month-to-month reduction of 0.48 OME (*p*<0.01) (Fig. 2a). There was a post-intervention level change of 35 more OME prescribed (*p*<0.01), followed by a post-intervention monthly reduction of 1.4 fewer OMEs per month (*p*<0.01) (Fig. 2a). Pre-ERAS total OME on the discharge prescription decreased by 2.26 OME per month (*p*<0.01), followed by a post-intervention level change of 51.5 fewer OMEs prescribed at hospital discharge (*p*<0.05) (Fig. 2b). Post-ERAS total OME on the discharge prescription continued to decrease by 3.78 OME per month (*p*<0.01) (Fig. 2b).

**Fig. 3:**
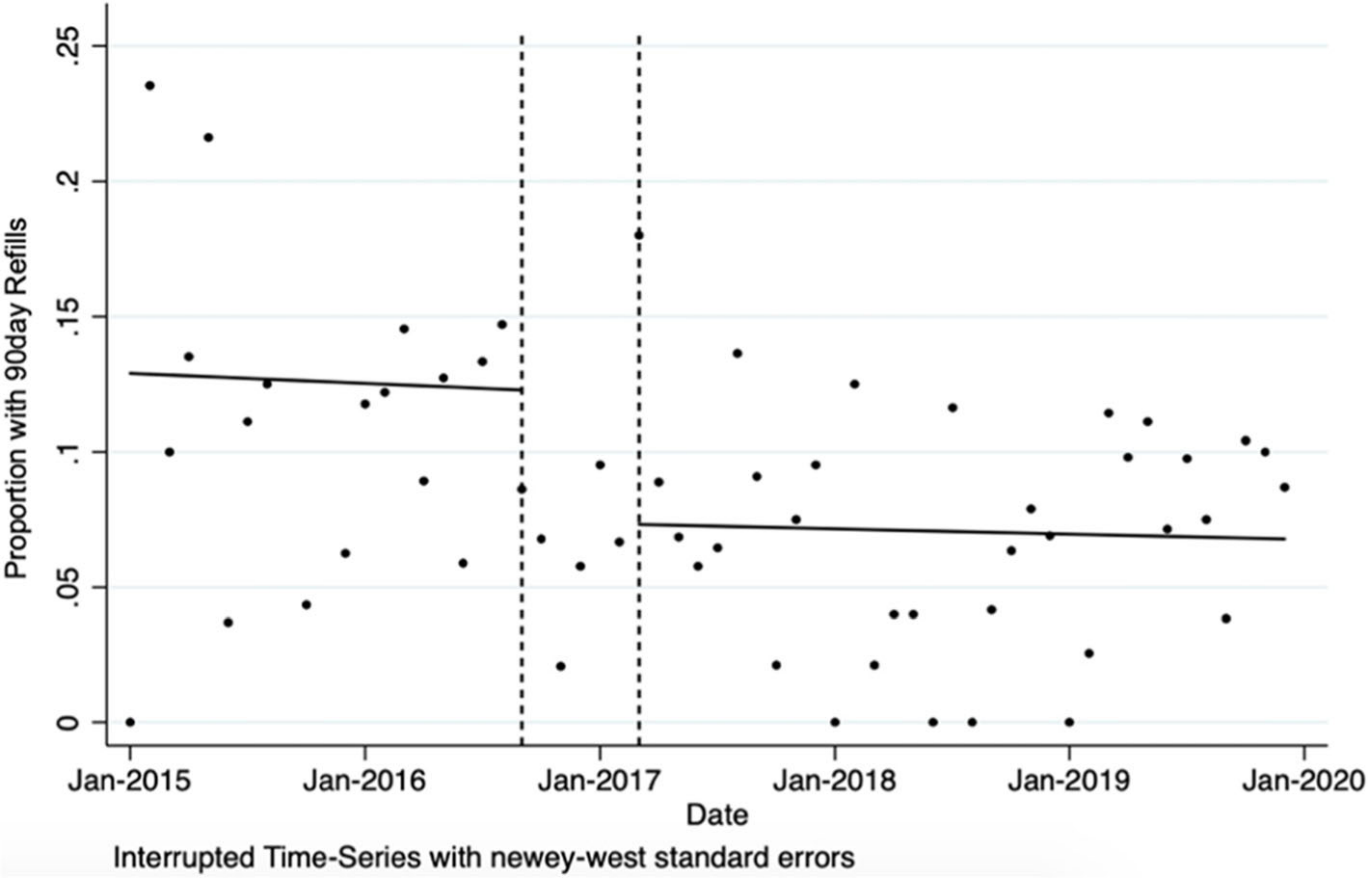
Change in the proportion of patients receiving an opioid refill prescription within 90 days after discharge, 2015–2019. Data are presented in monthly intervals on the *x*-axis. Vertical dashed lines represent the implementation phase. Post-ERAS implementation associated with a significant reduction of 4.96% (*p*=0.04).

**Fig. 4:**
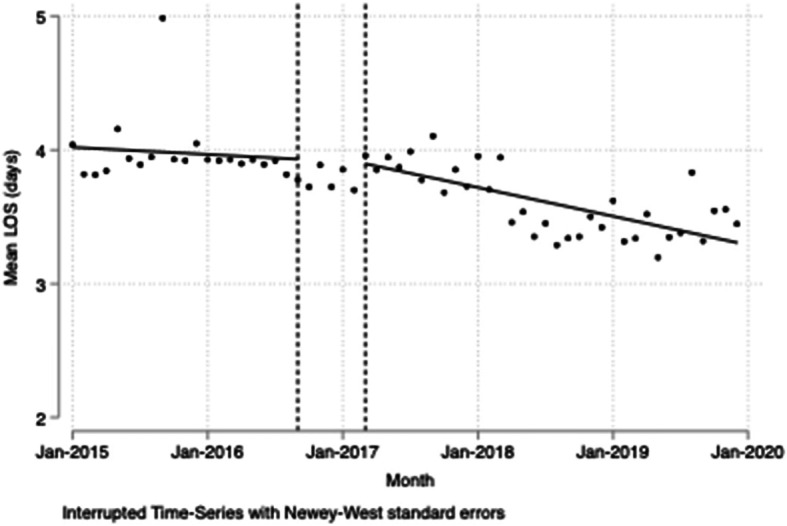
Change in postoperative length of stay, 2015–2019. Data are presented in monthly intervals on the *x*-axis and days on the *y*-axis. Vertical dashed lines represent the implementation phase. LOS= length of stay. Post-ERAS implementation was associated with a level change of − 0.03 days and slope change of − 0.02 days (*p*=0.76 and *p*=0.06, respectively)

Pre-ERAS, the proportion of patients receiving opioid refills decreased by 0.03% per month, followed by a post-intervention level change of 4.96% (*p*=0.04) (Fig. 3, Table 2). Post-ERAS opioid prescription refills followed a similar trend with a reduction of 0.02% per month that was not statistically significant compared to pre-intervention (*p*=0.85).

When comparing patients who received a discharge opioid prescription with those who did not, we found no significant differences in patient age, race, or length of stay (Tables 4 and 5 in Appendix). Pre-ERAS, patients who did not have an opioid prescribed at discharge were more likely to be opioid free the 24 h prior to discharge compared to patients who did receive a discharge opioid prescription (88.9% vs. 17.7%, *p*<0.01) (Table 4 in Appendix). Similarly, post-ERAS patients who did not have an opioid prescribed at discharge were more likely to be opioid free in the 24 h prior to discharge compared to patients who did receive a discharge opioid prescription (91.2% vs. 39.8%, *p*<0.01) (Table 5 in Appendix).

## Discussion

Consistent with prior studies examining the effects of ERAS pathways, our study found a variety of benefits associated with the implementation of pain management guidelines at our institution for patients undergoing cesarean delivery (Shinnick et al. 2020). These benefits included an increase in the adoption of inpatient multimodal analgesia and a reduction in inpatient opioid consumption. In addition, there was a reduction in the proportion of patients receiving a discharge opioid prescription and a reduction in opioid refills in the first 90 days after discharge.

Using ITSA, we were able to identify nuanced temporal prescribing changes that have not been captured in prior ERAS cesarean cohort analyses. Our institution’s ERAS protocol, which is intended to reduce opioid overprescribing, was paradoxically associated with a significant increase in the strength (daily OMEs) of the discharge opioid prescriptions. This finding suggests that implementation of the ERAS protocol may have inadvertently increased the daily dose of opioid prescribed to patients who received an opioid prescription. The largest increase in daily OMEs on the discharge prescription occurred immediately after the implementation phase and steadily decreased over time. This is concerning given that increases in daily OMEs on discharge prescriptions are associated with increases in misuse or overdose events (Dowell et al. 2016). Furthermore, the post-ERAS cohort had a higher rate of discharge prescriptions exceeding 90 daily OMEs. This increase in high-risk prescriptions was accompanied by a decrease in total OMEs on the discharge prescription after ERAS implementation. Our results suggest that the ERAS pathway was associated with a higher daily OME written on the discharge prescription, but for a shorter duration. In addition, we found a significant change in opioid type to oxycodone. These findings directly reflect the ERAS protocol, which limited prescriptions to 20 pills and defined the maximum dose of 10 mg of oxycodone every 4 h, which translates to 90 daily OMEs. Based on our results, we believe that when prescribers are provided a dosing range to choose from, there is a risk of defaulting to the maximum dose recommended, and this finding should be considered when designing pain medication order sets for implementation in the EHR.

The potential effects that opioid dose or duration limits have on physician opioid prescribing behavior remains variable (Agarwal et al. 2019a; Echeverria-Villalobos et al. 2020). A multitude of factors affect prescribing behaviors, including diagnostic skills, clinical judgement, drug knowledge, financial incentives, and motivation to remain up to date on medical practices, which are variable among practitioners (Stern and Trajtenberg 1998). It is possible that our ERAS protocol nudged physicians into prescribing in the higher range of the suggested discharge opioid prescription resulting in a higher daily OME in the early post-ERAS time period. Drivers for this behavior were not explored in our study, but may include concerns regarding patient need for refills and ensuring patient satisfaction. It is possible that prescribers chose to prescribe higher opioid doses to ensure adequate pain control after ERAS guidelines recommended an upper limit on the number of pills prescribed.

Our findings highlight important and modifiable consequences with ERAS implementation. First, there is a need for data collection and evaluation at early phases of policy implementation to identify the effects of protocolization of prescribing behaviors. It may be reasonable to deploy small pilot studies to help identify and mitigate any unintended consequences prior to rolling out new policies on larger cohorts. Second, protocolization of care may prove beneficial for inpatient pain management, but might be problematic for discharge opioid prescriptions. Other studies have emphasized the benefits of individualized or patient-tailored prescribing practices to reducing excessive opioid prescribing instead of relying on a one-size fits all approach (Hill et al. 2018; Agarwal et al. 2019b). Consistent with the recommendation by Levy et al. in their international consensus statement on preventing opioid-related harm in adult surgical patients, a patient-centered approach should be utilized when determining the discharge opioid prescription to reduce over prescribing after surgery (Levy et al. 2021). Initiatives to develop and modify ERAS protocols should balance the goals of safe opioid prescribing in surgical patients and avoidance of inadvertent harm arising from these policies while ensuring that individual patients’ pain management needs are adequately managed during the inpatient stay as well as post-discharge.

## Limitations

Our study was conducted during a time of increasing evidence of harm associated with unsafe opioid prescribing practices along with the introduction of national- and state-level opioid prescribing initiatives, which may have contributed to the changes in opioid prescribing practices we found. Nationally, the CDC published guidelines for prescribing opioids for chronic pain (Dowell et al. 2016). However, these guidelines were intended for chronic pain patients, not for acute postoperative opioid naïve patients. The state of California, where our study is located, did not pass any state-specific legislation to limit opioid prescriptions during the study period. However, California did update its prescription drug monitoring programs (PDMP) policy in July 2016 to require mandatory registration of opioid prescribers, when previously this was voluntary. Regardless, mandatory PDMP registration has not been shown to significantly reduce opioid prescriptions for surgical patients (Stucke et al. 2018).

In addition, our data is from a single large, tertiary, academic medical center and our observations may not be generalizable to patients undergoing cesarean delivery or other surgical procedures at non-academic centers or in other regions of the country. The retrospective observational study design using electronic health records limits our ability to assert a direct causal relationship between the explanatory factors in our model and opioid prescribing patterns. It is possible that factors that were unaccounted for in our model may contribute to the observed findings if these occurred at the same time as ERAS implementation. It is also important to note that ITSA models assume a linear relationship to estimate the change in the outcomes over time during the pre- and post-ERAS implementation phases, but more nuanced relationships may exist. In addition, our data was unable to account for inpatient administration of acetaminophen that was in combination with an opioid (i.e., hydrocodone-acetaminophen, oxycodone-acetaminophen). As a result, pre-ERAS acetaminophen administration prior to discharge may be underestimated. Finally, our data captured discharge prescriptions written for patients but does not capture if the prescription was filled nor how much opioid was actually taken by the patient. Therefore, patients may not have been exposed to the doses of opioids prescribed at hospital discharge. Despite these limitations, our study highlights the importance of longitudinal evaluation of opioid prescribing practices after new guidelines are implemented to understand whether they are having the intended effect and to mitigate against perpetuating high-risk opioid prescribing practices.

## Conclusions

In this study, we identified unintended consequences of provider opioid prescribing patterns in the immediate post-ERAS implementation period for patients undergoing cesarean delivery at a large tertiary academic medical center. While ERAS implementation led to an increase in multimodal analgesia and opioid-free pain control in the last 24 h prior to discharge, we also found a significant increase in the daily OME written on the discharge opioid prescription. These findings highlight the importance of early and continued evaluation after the implementation of new policies that are intended to reduce the risk of opioid exposure in selected patients.

## Data Availability

Raw large-scale electronic medical record data were generated at our institution. Derived data supporting the findings of this study are available from the corresponding author on reasonable request with permission of IRB.

## Appendix

**Table 3:** Multivariable ITSA model for the association of daily oral morphine equivalents on discharge prescription with ERAS

	Coefficient	95% CI low	95% CI high	***P*** value
Pre-ERAS trend (Jan 2015–Sep 2016)	− 0.53	− 0.75	− 0.32	<0.01
Level Shift after ERAS implementation	34.93	29.63	40.22	<0.01
Trend Shift after ERAS implementation	− 0.77	− 1.11	− 0.43	<0.01
Provider type	3.55	− 12.17	19.28	0.65
Age	1.57	− 0.69	3.82	0.17
Postoperative length of stay	5.76	− 3.09	14.62	0.20

**Table 4:** Clinical and demographic characteristics of pre-ERAS cohort by receipt of opioid prescription at discharge

	Opioid prescribed (*n*=740)	No opioid prescribed (*n*=36)	*P* value
Age, mean (SD)	34.06 (5.15)	33.83 (5.54)	0.79
Race, *n* (%)			
White or Caucasian	328 (44.32)	19 (52.78)	0.15
Asian	169 (22.84)	11 (30.56)	
Black or African American	48 (6.49)	1 (2.78)	
Native Hawaiian or Other Pacific Islander	16 (21.62)	0 (0)	
American Indian or Alaska Native	4 (0.54)	0 (0)	
Unknown	175 (23.65)	5 (13.89)	
Opioid-free 24 h pre-discharge, *n* (%)	131 (17.70)	32 (88.89)	<0.01
Length of stay in days, mean (SD)	3.97 (1.34)	3.82 (0.72)	0.51

**Table 5:** Clinical and demographic characteristics of post-ERAS cohort by receipt of opioid prescription at discharge

	Opioid prescribed (*n*=1188)	No opioid prescribed (*n*= 285)	*P* value
Age, mean (SD)	35.56 (5.488)	34.92 (5.38)	0.08
Race, *n* (%)			0.24
White or Caucasian	549 (46.21)	128 (44.91)	
Asian	256 (21.55)	79 (27.72)	
Black or African American	73 (6.14)	14 (4.91)	
Native Hawaiian or Other Pacific Islander	17 (1.43)	2 (0.7)	
American Indian or Alaska Native	8 (0.67)	0 (0.0)	
Unknown	285 (23.99)	62 (21.75)	
Opioid-free 24 h pre-discharge, *n* (%)	473 (39.81%)	260 (91.22%)	<0.01
Length of stay in days, mean (SD)	3.65 (1.50)	3.63 (0.99)	0.83

## Abbreviations

ERAS: Enhanced Recovery After Surgery
OME: Oral morphine equivalent
NSAID: Non-steroidal anti-inflammatory drug
